# Developing a prediction model to identify people with severe mental illness without regular contact to their GP - a study based on data from the Danish national registers

**DOI:** 10.1186/s12888-024-05743-x

**Published:** 2024-04-23

**Authors:** Astrid Helene Deleuran Naesager, Sofie Norgil Damgaard, Maarten Pieter Rozing, Volkert Siersma, Anne Møller, Katrine Tranberg

**Affiliations:** 1 Department of Public Health, The Section of General Practice and the Research Unit for General Practice, University of Copenhagen, Copenhagen, Denmark; 2 Psychiatric Center Copenhagen, Copenhagen, Denmark; 3 Section of General Practice, The Research Unit for General Practice, Department of Public Health, University of Copenhagen, Øster Farimagsgade 5, DK-1014 Copenhagen, Region Zealand Denmark

**Keywords:** Severe mental disorder, General practitioner, Patient care continuity, Health care, Logistic models, ROC curve

## Abstract

**Introduction:**

People with severe mental illness (SMI) face a higher risk of premature mortality due to physical morbidity compared to the general population. Establishing regular contact with a general practitioner (GP) can mitigate this risk, yet barriers to healthcare access persist. Population initiatives to overcome these barriers require efficient identification of those persons in need.

**Objective:**

To develop a predictive model to identify persons with SMI not attending a GP regularly.

**Method:**

For individuals with psychotic disorder, bipolar disorder, or severe depression between 2011 and 2016 (*n* = 48,804), GP contacts from 2016 to 2018 were retrieved. Two logistic regression models using demographic and clinical data from Danish national registers predicted severe mental illness without GP contact. Model 1 retained significant main effect variables, while Model 2 included significant bivariate interactions. Goodness-of-fit and discriminating ability were evaluated using Hosmer-Lemeshow (HL) test and area under the receiver operating characteristic curve (AUC), respectively, via cross-validation.

**Results:**

The simple model retained 11 main effects, while the expanded model included 13 main effects and 10 bivariate interactions after backward elimination. HL tests were non-significant for both models (*p* = 0.50 for the simple model and *p* = 0.68 for the extended model). Their respective AUC values were 0.789 and 0.790.

**Conclusion:**

Leveraging Danish national register data, we developed two predictive models to identify SMI individuals without GP contact. The extended model had slightly better model performance than the simple model. Our study may help to identify persons with SMI not engaging with primary care which could enhance health and treatment outcomes in this group.

**Supplementary Information:**

The online version contains supplementary material available at 10.1186/s12888-024-05743-x.

## Introduction

In Denmark, 580,000 people have been diagnosed with a severe mental illness (SMI), defined as psychotic disorders and severe affective disorders (bipolar disorder, and severe unipolar depression) in 2023 [1, 2]. Globally, 970 million people were living with a mental illness in 2019, of which 2.5% had schizophrenia, 4.1% had bipolar disorder and 28.9% had a depressive disorder [3]. SMI has serious health implications and has been associated with a shorter life expectancy of up to 20 years compared to the general population [3]. The lower life expectancy is likely attributable to the higher prevalence of somatic diseases, such as cancer, cardiovascular disease, and type 2 diabetes mellitus [1, 4–6].

In most government-funded healthcare systems, the general practitioner (GP) functions as a gatekeeper to the secondary healthcare system. Moreover, the GP has the responsibility of identifying and treating somatic diseases and also referring some persons to specialized treatment in the secondary health care system [7–9]. Research in the general population has shown, that continuity of care with a regular GP has a beneficial impact on disease trajectories and mortality in the general population [9]. Therefore, regular contact with a GP might promote the prevention and timely treatment of somatic health problems in individuals with SMI.

In line with the higher somatic mortality, previous studies have found that persons with SMI tend to have a higher contact frequency with GP compared to the general population [10–12]. However, reaching people in greatest need of new initiatives and interventions in different healthcare settings has shown to be unequally distributed [13] and a study have shown that people with SMI in general have a lower socioeconomic position, and are less likely to be identified, approached, and participate in trials and that the recruitment strategy for this population should be considered carefully [14]. In addition to this another study discovered, that healthcare providers also encounter difficulties in identifying persons with SMI with unidentified or poor management of somatic disease [15]. Potential initiatives such as an outreach service by GP could promote patient engagement and improve treatment and prevention of somatic illness in this group. However, such initiatives would require efficient identification of persons in most need. To facilitate the identification of persons at risk of somatic disease, there is a growing interest in prediction models [16]. These models often use biological measures, however such models could benefit from including sociodemographic variables in their prediction [17]. Previous studies have explored which sociodemographic factors that characterize persons with SMI with and without contact with a GP. However, to our knowledge, no studies have aimed to create a prediction model for contact with GP for people with SMI based on sociodemographic factors. The objective of this study was therefore to fill the existing gap in the literature by developing a prediction model to identify persons with SMI without contact with GP by using data from the Danish national registers.

## Method

### Study design

**Fig. 1:**
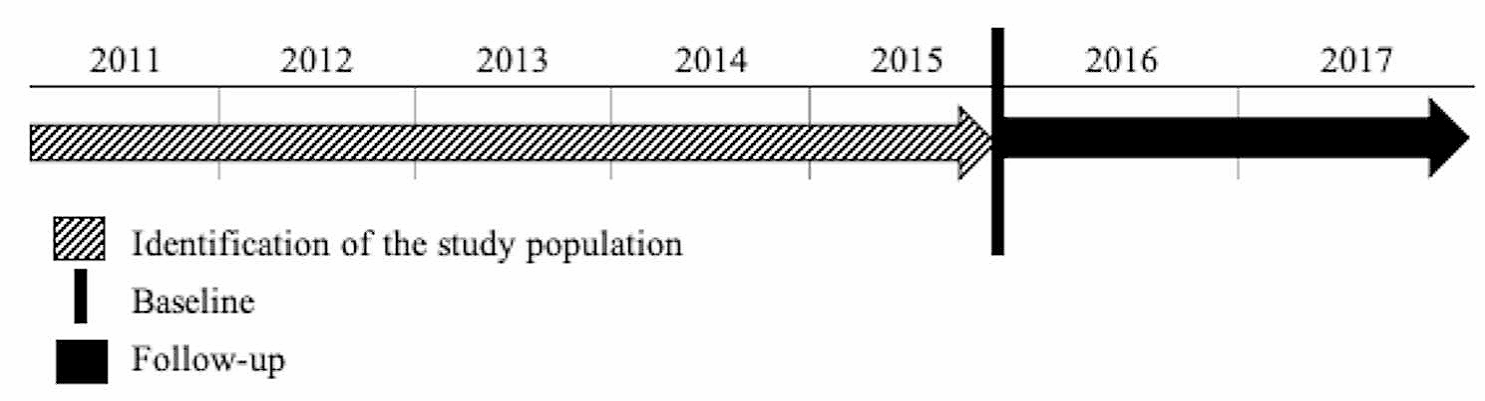
Study design

We have designed a prediction model for no contact with GP based on a cohort of persons with SMI, where information on the study population was collected retrospectively. Data was collected from nationwide registers and merged by the Civil Registration Number (CPR number). The study population included those persons registered with contacts in secondary care due to SMI in the period 2011–2015. For these persons, the number of contacts with GP was recorded over the period 2016–2017 (Fig. 1).

### Registers

The study population was identified using the Danish Psychiatric Central Register (DNPR-Psych), a sub-register of the Danish National Patient Register (DNPR). These registers contain information on all public and private hospital sector contacts, and the DNPR is one of the oldest nationwide hospital registers globally [18, 19]. The DNPR was also used for information on *comorbidities*.

The Danish National Health Service Register for Primary Care (NHSR) [20] was used to gather data on *GP contacts* and *Out-of-hours contacts.* The register contains information about the activities of health professionals in the public healthcare system, such as general practitioners, psychologists, and physiotherapists. Each contact is registered with a CPR number [20].

The Civil Registration System (CPRS) is a nationwide register for all Danish citizens, where each person is assigned a CPR number. The CPR number allows for linking data across registers and IT systems [21]. Additionally, the register includes information on demographic variables such as *sex*, *age*, *ethnicity*, *marital status*, and *region of living*.

The Danish Education Register (DER), Income Statistics Register (ISR), Employment Classification Module (ECM), Danish Register of Causes of Death, and the Danish Prevention Register were also used to collect information on demographic variables, as well as information about the time of death and migration out of Denmark during the study period [22–26].

### Study population

**Fig. 2:**
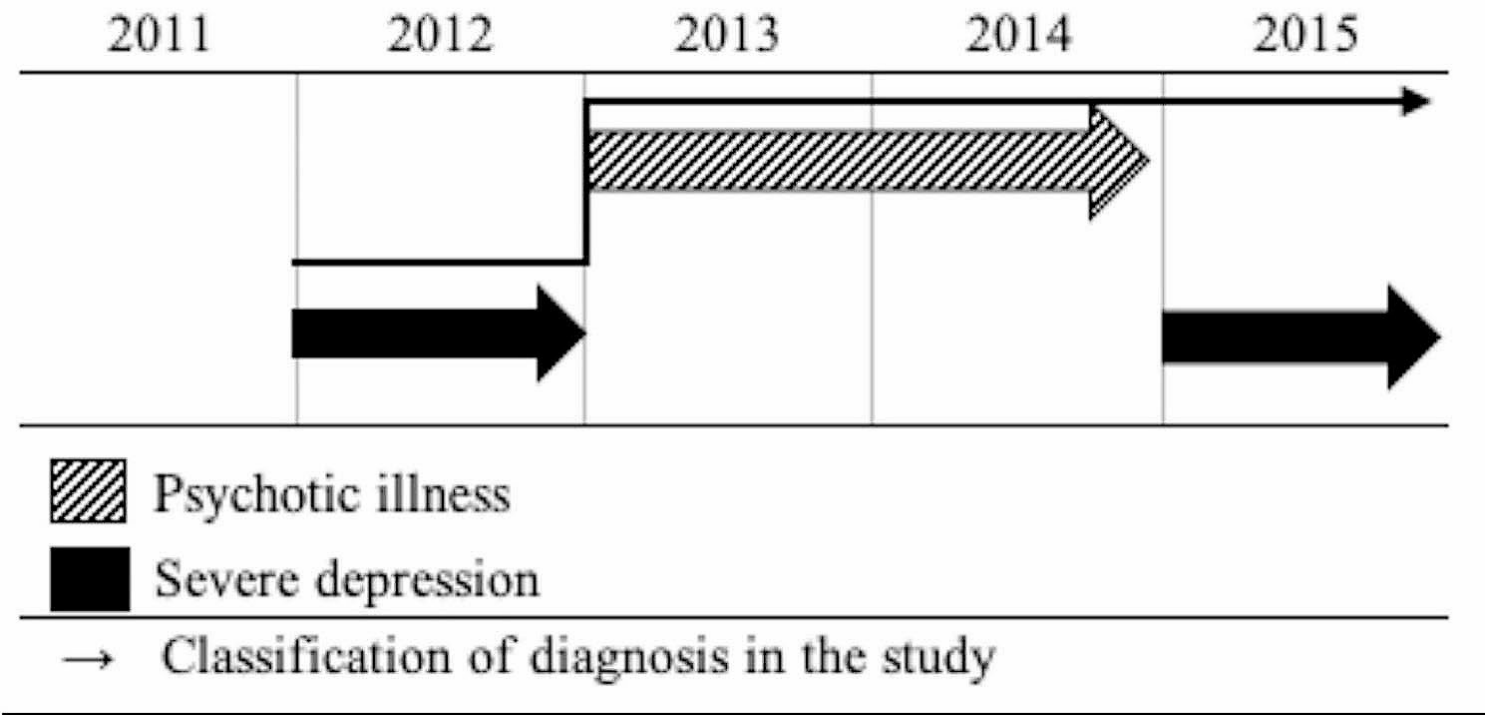
Diagnosis hierarchy

**Fig. 3:**
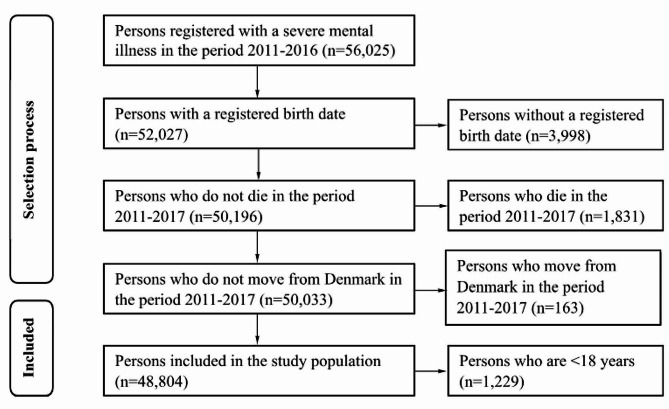
Flowchart of the study population

Our study population included adults above 18 years of age registered in the DNPR-Psych with an SMI from the International Classification of Diseases Tenth Revision (ICD-10) classification system [27] defined as psychotic illness (F20-F29), bipolar disorder (F31), or severe depression (F32.2, F32.3) as a main diagnosis in the inclusion period. If a person was diagnosed with multiple SMI diagnoses, a hierarchy was applied. Persons with multiple SMI diagnoses were allocated to the diagnostic group with the greatest disease severity. The hierarchy is illustrated in Fig. 2. Persons who did not have a valid birthdate or CPR number, died, or moved out of the country between 2011 and 2017 were excluded. The final study population consisted of 48,804 persons. A flow chart of the study population selection is depicted in Fig. 3.

### Outcome

The outcome was the total number of contacts to GP in the follow-up period for each person in the study population. Data were derived from the NHSR and included all services in GP during the opening hours, including contact by telephone and email. We excluded out-of-hours contacts. “No contact” was defined as less than three contacts with GP in the two-year follow-up period. The threshold was set after considering the overall distribution of contact with GP in the study population.

### Other variables

Demographic data (*age, sex, marital status, ethnicity, region*) were obtained from CPRS at baseline. Information on *education, income*, and *occupation* were obtained from DER, ISR, and ECM at baseline. *Comorbidity* was measured using a modified version of the Quality and Outcomes Framework (QOF) (Supplementary file 1). QOF is a simple measure that counts and summarizes the number of comorbidities for each individual based on a list of frequent chronic diseases that are particularly treated in general practice [28]. Data on *comorbidity* was obtained from DNPR in the inclusion period and divided into categories of 0,1 or 2 or more comorbidities.

In the follow-up period data on contact with additional health services was obtained from NHSR, DNPR, and DNPR-Psych and divided into three variables: *Out-of-hours contacts with GP, contacts to secondary services (hospital)*, and *contacts to psychiatric services.*

See supplementary file 2 for a list of all the included variables.

### Model performance

Receiver Operating Characteristic (ROC) curves are graphical plots illustrating the models’ performance conditional to different thresholds of the probability of the outcome [29]. The ROC curves therefore illustrate the models’ ability to predict and differentiate between the binary outcome at varying thresholds [30]. The area under the ROC curve (AUC) is an overall measure of the model’s discriminative ability. A perfect discriminative ability is indicated by AUC = 1 and a value of AUC = 0.5 indicates that the discriminative ability is not better than chance.

A concordance statistic (ROC curves) and goodness-of-fit statistic (Hosmer-Lemeshow (HL) test) were calculated to evaluate the discriminative performance and calibration of the models. The HL test is a goodness-of-fit test that assesses the conformity between the model’s predicted probability of the outcome, and the actual probability of the outcome in the data [29, 31]. The outcome of the test is a p-value. A significant p-value indicates that the model’s prediction differs significantly from the observed values, indicating that the model has an insufficient calibration.

### Statistical analysis

The statistical analyses were conducted in SAS version 9.4. Chi-square tests (categorical variables) and general linear model (continuous variables) were used to assess differences in the baseline characteristics across diagnoses of the study population at baseline.

Multivariable logistic regression analysis with a backward step-by-step approach was conducted to develop the model structures of two prediction models for no contact with general practice. Hence, influential variables to be included in the predictive models were identified through significance tests in these models, where the only implicit assumption of consequence is of linearity in the logit for continuous variables [32]. A simple model included the demographic and contact with additional health services variables. An extended model included both demographic and contact with additional health services variables and two-way interactions between these. All 14 demographic and contact with additional health services variables were included in both models, and all possible two-way interactions was also included in the extended model as a starting point. Thereafter, incrementally, the variable with the highest p-value was removed, until all variables included in the simple model and all variables or interactions in the extended model had a p-value < 0.001. For the extended model, insignificant variables remained in the model if they were part of a significant two-way interaction.

The parameter estimates of the two models were found by subsequently carrying out the models on a random selection of 90% of the study population. The two models that were developed were a simple model that included only significant variables and an extended model that included significant variables and significant two-way interactions (*p* < 0.001).

Finally, the models were cross-validated on the remaining 10% of the study population according to the hold-out method. Hence, the performance test on the 10% holdout data of the models, found through the model search and the belonging parameters estimated in the 90% holdout data, was assessed through the AUC of the two ROC curves (discrimination) and the HL test (calibration).

**Fig. 4:**
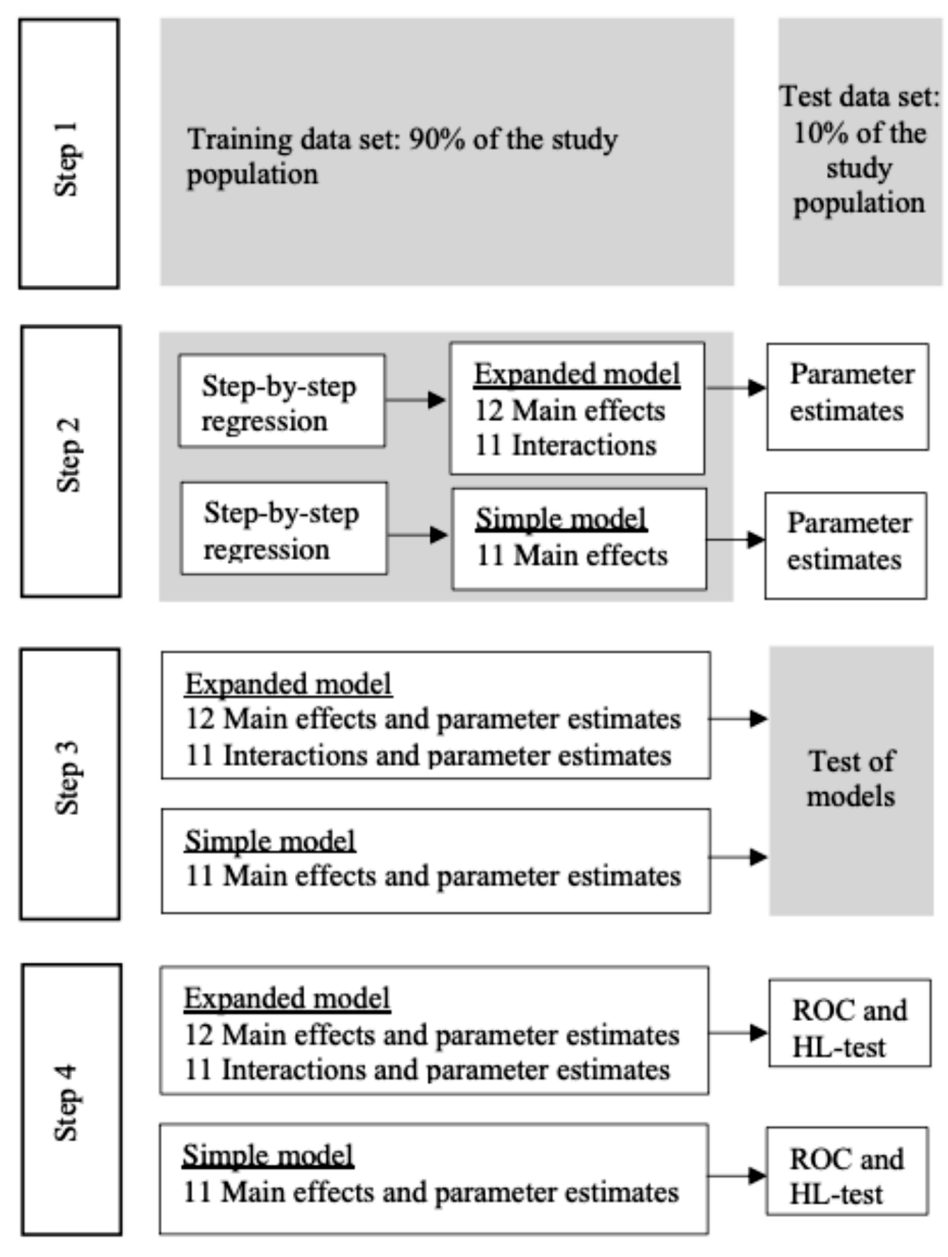
Procedure for cross-validation

The procedure for the cross-validation is displayed in Fig. 4.

## Results

### Descriptive results

Table 1 shows the distribution of the included variables in the study population and across the SMI (diagnoses) groups at baseline. The mean age was 43.5 years and 50.0% were women. The distribution of all variables differed significantly between the SMI diagnoses at baseline (*p* < 0.001). People with high odds of 0–2 contacts with GP were characterized by having a psychotic disorder, being male, living in region Zealand, being of other origin than Danish, being unmarried, having a low income, not having any comorbidities, and having high contact with psychiatric services.

8.9% of the study population had 2 or fewer contacts with a GP in the follow-up period. Persons with psychotic disorders had the highest proportion of persons with 0–2 contacts with GP, and persons with bipolar disorder had the lowest proportion with 0–2 contacts (*p* < 0.001). A sensitivity analysis using a cut-off point at < 7 contacts to GP showed only marginal changes in the associations. 79.6% of the study population had ≥ 7 contacts (Supplementary file 3).

**Table 1:** Characteristics of the study population

	Total *n* = 48,804 (100)	Psychotic illness *n* = 31,590 (64.7)	Bipolar illness *n* = 10,977 (22.5)	Severe depression *n* = 6237 (12.8)	p-value
**Age (SD)**	43.5 (16.3)	41.6 (15.7)	47.8 (16.1)	45.8 (17.7)	< 0.0001*
**Age categories**					
18–29 years	12,285 (25.2)	9189 (29.1)	1707 (15.6)	1389 (22.3)	< 0.0001*
30–39 years	9420 (19.3)	6342 (20.1)	2035 (18.5)	1043 (16.7)	
40–49 years	9787 (20.1)	6258 (19.8)	2259 (20.6)	1270 (20.4)	
50–59 years	8619 (17.7)	5285 (16.7)	2205 (20.1)	1129 (18.1)	
60–69 years	5207 (10.7)	2889 (9.2)	1601 (14.6)	717 (11.5)	
70 + years	3486 (7.1)	1627 (5.2)	1170 (10.7)	689 (11.1)	
**Sex**					
Male	24,403 (50.0)	17,524 (55.5)	4247 (38.7)	2632 (42.2)	< 0.0001*
Female	24,401 (50.0)	14,066 (44.5)	6730 (61.3)	3605 (57.8)	
**Region**					
Northern Jutland	3872 (7.9)	2378 (7.5)	1003 (9.1)	491 (7.9)	< 0.0001*
Mid Jutland	10,903 (22.3)	5849 (18.5)	3326 (30.3)	1728 (27.7)	
Southern Denmark	10,792 (22.1)	7113 (22.5)	2294 (20.9)	1385 (22.2)	
Capitol City	16,922 (34.7)	11,951 (37.8)	3078 (28.0)	1893 (30.4)	
Zealand	6315 (12.9)	4299 (13.6)	1276 (11.6)	740 (11.9)	
**Ethnicity**					
Danish	41,688 (85.4)	26,403 (83.6)	10,121 (92.2)	5164 (82.8)	< 0.0001*
Immigrants and descendants	7116 (14.6)	5187 (16.4)	856 (7.8)	1073 (17.2)	
**Marital status**					
Married/in a relationship	9451 (19.4)	3965 (12.6)	3221 (29.3)	2256 (36.3)	< 0.0001*
Not married	29,116 (59.7)	22,203 (70.3)	4469 (40.7)	2444 (39.2)	
Divorced	8596 (17.6)	4667 (14.8)	2800 (25.5)	1129 (18.1)	
Widow	1641 (3.4)	755 (2.4)	487 (4.4)	399 (6.4)	
Occupation**					
Working	6890 (14.1)	2617 (8.3)	2364 (21.5)	1909 (30.6)	< 0.0001*
Without work	36,226 (74.2)	26,221 (83.0)	6701 (61.1)	3304 (53.0)	
Retired	5685 (11.7)	2749 (8.7)	1912 (17.4)	1024 (16.4)	
Income***					
< 150,000 DKK.	17,441 (35.7)	12,336 (39.1)	3131 (28.5)	1974 (31.7)	< 0.0001*
150,001-225,000 DKK.	22,070 (45.2)	15,240 (48.2)	4639 (42.3)	2191 (35.1)	
225,001-300,000 DKK.	5992 (12.3)	2883 (9.1)	1981 (18.1)	1128 (18.1)	
> 300,000 DKK.	3298 (6.8)	1128 (3.6)	1226 (11.2)	944 (15.1)	
Education****					
Short	24,478 (50.2)	18,436 (58.4)	3609 (32.9)	2433 (39.0)	< 0.0001*
Intermediate	16,169 (33.1)	9384 (29.7)	4354 (39.7)	2431 (39.0)	
Long	7951 (16.3)	3633 (11.5)	2971 (27.1)	1347 (21.6)	
**Comorbidity**					
0	37,457 (76.8)	24,756 (78.4)	8055 (73.4)	4646 (74.5)	< 0.0001*
1	7845 (16.1)	4818 (15.3)	1942 (17.7)	1085 (17.4)	
≧ 2	3502 (7.2)	2016 (6.4)	980 (8.9)	506 (8.1)	
**Contact with general practice**					
Contact (> 2)	44,440 (91.1)	27,983 (88.6)	10,574 (96.3)	5883 (94.3)	< 0.0001*
No contact (≤ 2)	4364 (8.9)	3607 (11.4)	403 (3.7)	354 (5.7)	
**Out-of-hours contacts**					
0	36,083 (73.9)	23,176 (73.4)	8182 (74.5)	4725 (75.8)	< 0.0001*
1–2	4723 (9.7)	2975 (9.4)	1145 (10.4)	603 (9.7)	
3–6	4192 (8.6)	2701 (8.6)	935 (8.5)	556 (8.9)	
≧ 7	3806 (7.8)	2738 (8.7)	715 (6.5)	353 (5.7)	
**Contact with secondary services (hospital)**					
0	15,339 (31.4)	10,924 (34.6)	2819 (25.7)	1596 (25.6)	< 0.0001*
1–2	10,855 (22.2)	6569 (20.8)	2620 (23.9)	1666 (26.7)	
3–6	12,720 (26.1)	7681 (24.3)	3209 (29.2)	1830 (29.3)	
≧ 7	9890 (20.3)	6416 (20.3)	2329 (21.2)	1145 (18.4)	
**Contact with psychiatric services**					
0	24,094 (49.4)	13,781 (43.6)	5960 (54.3)	4353 (69.8)	< 0.0001*
1–2	12,432 (25.5)	8594 (27.2)	2726 (24.8)	1111 (17.8)	
3–6	7493 (15.4)	5403 (17.1)	1524 (13.9)	566 (9.1)	
≧ 7	4786 (9.8)	3812 (12.1)	767 (7.0)	207 (3.3)	

*1% significance level

**missing: 3

***missing: 3

**** missing: 206

### Prediction models for 0–2 contacts with GP

**Fig. 5:**
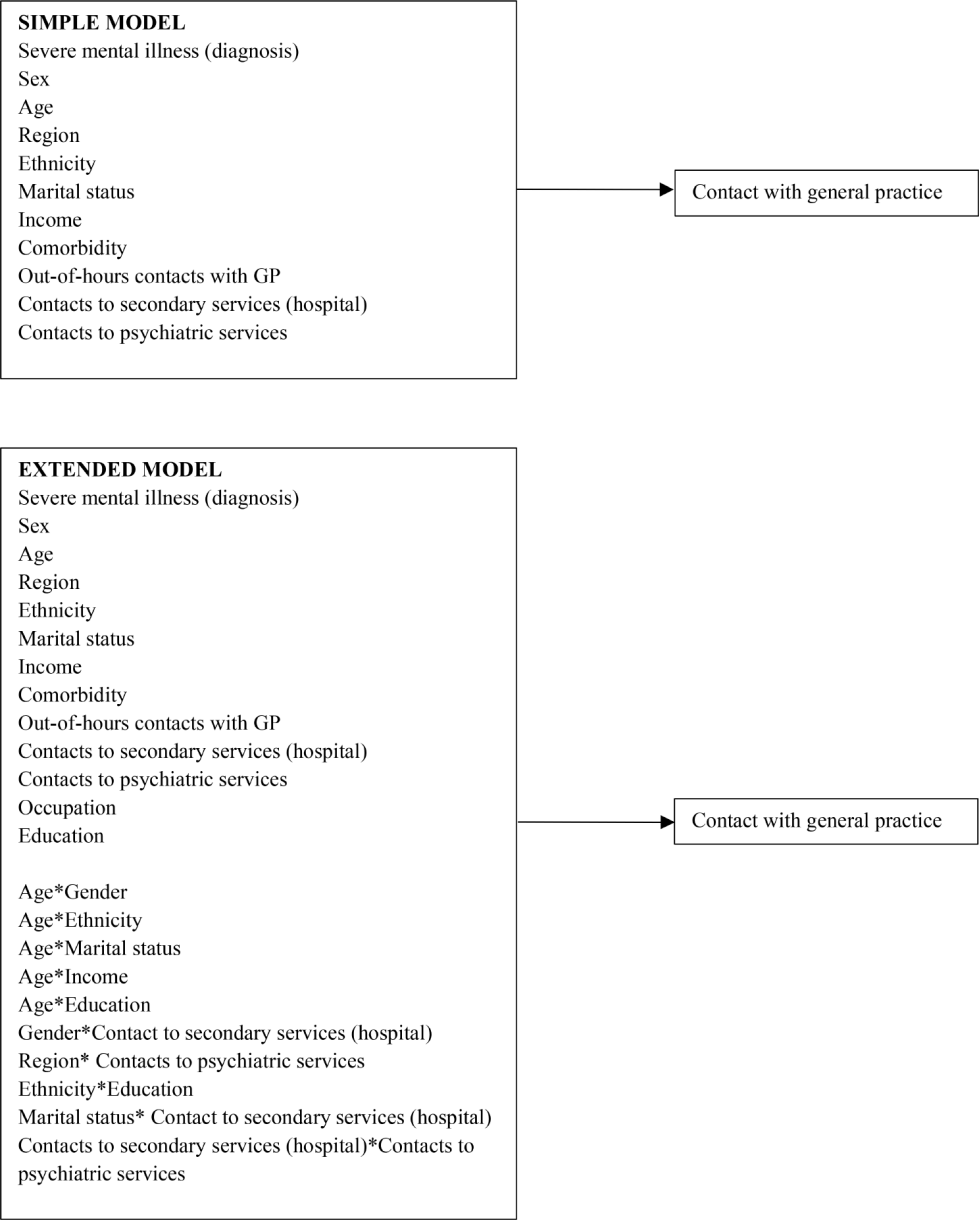
Model structure for the simple and extended model

The simple logistic model based on a random sample of 90% of the study population yielded a model including the variables *SMI, sex, age, region, ethnicity, marital status, income, comorbidity, out-of-hours contacts with GP, contacts to secondary services (hospital)* and *contacts to psychiatric services*, which all had a significant association with 0–2 contacts witch GP (see Supplementary file 4). The extended logistic model yielded a model, that included the same variables as the simple model as well as *occupation, education*, and ten two-way interactions (Fig. 5).

**Fig. 6:**
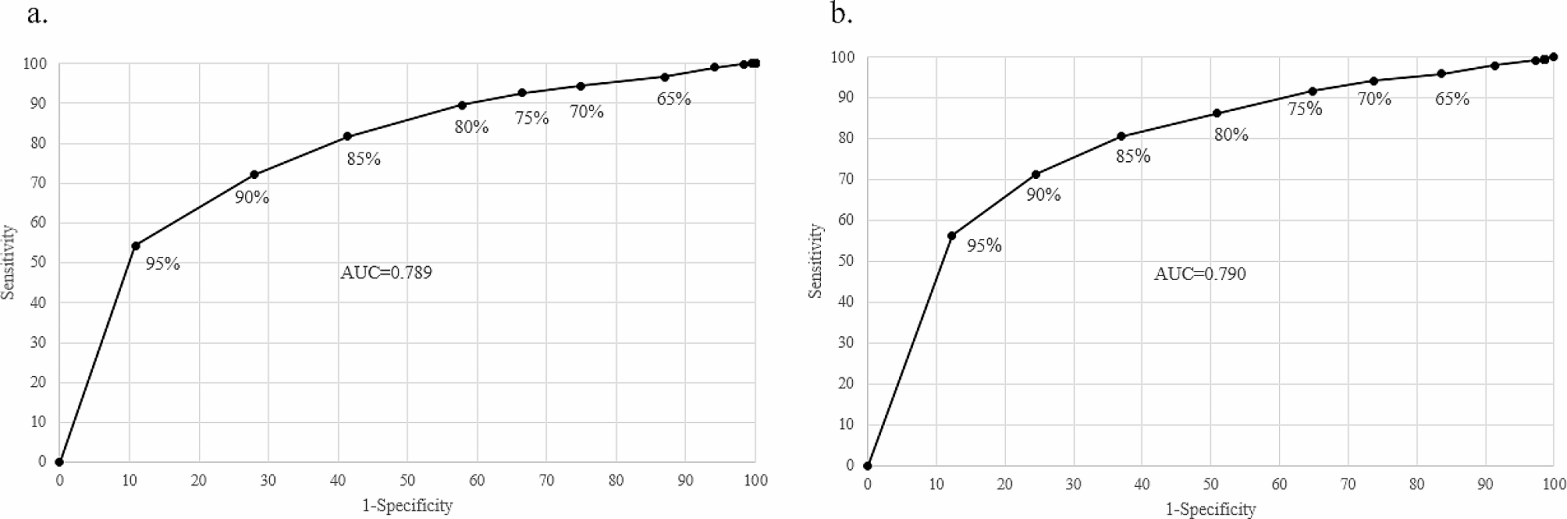
ROC curves for the simple and extended model. **a** ROC curve for the simple model. **b** ROC curve for the extended model

Receiver Operating Characteristic (ROC) curves were applied to assess the prediction models’ discriminative ability. The curves show the models’ ability to classify the outcome correctly conditional on consecutive thresholds [29], and are presented in Fig. 6. The overall discriminative ability of the models are measured by the AUC, where perfect discriminative ability is indicated by AUC = 1. The %-points on the curves represent the threshold for when the model classifies an observation as an outcome. The simple model had an AUC of 0.789 and the extended model had an AUC of 0.790.

**Fig. 7:**
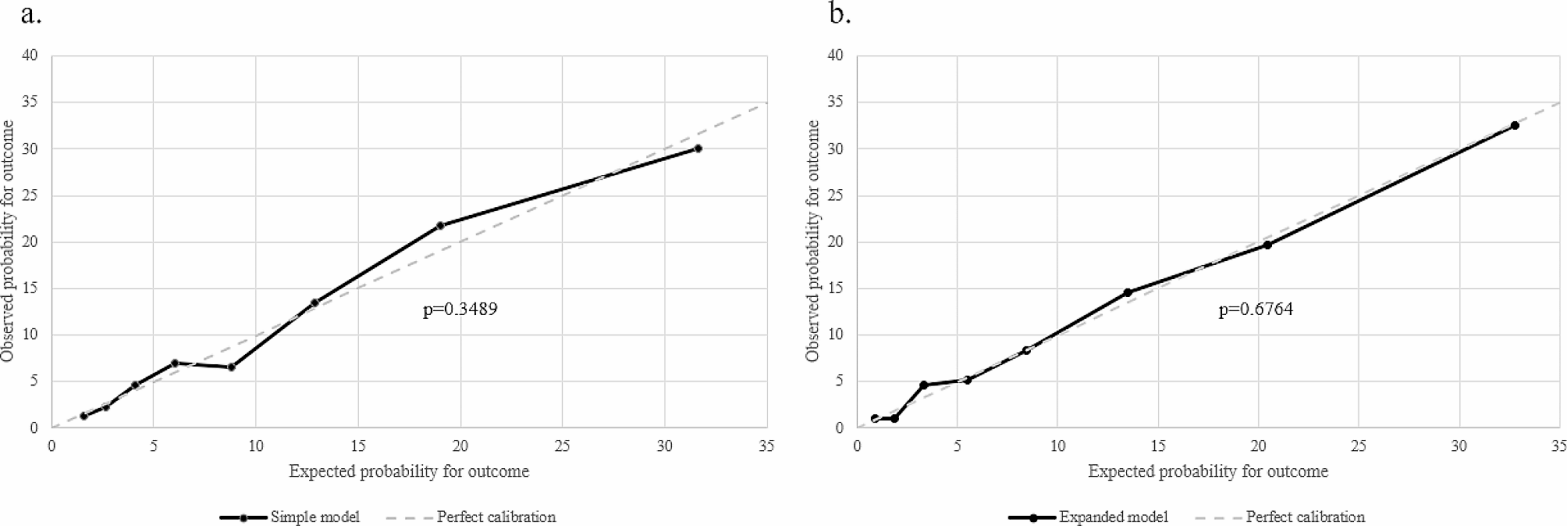
Calibration (HL test) for the Simple and extended model. **a** Calibration for the simple model. **b** Calibration for the extended model

The goodness-of-fit test (HL test) was applied to assess the performance of the prediction models in terms of calibration that is, the conformity between the model’s predicted probability of the outcome, and the actual probability of the outcome observed in the data [29, 31]. The results of the tests are presented in Fig. 7. The calibration curves show the correspondence between the observed and expected probability for the outcome. In the figure, the dotted line indicates perfect calibration, where the expected probabilities for the outcome predicted by the model, correspond to the observed probabilities. The p-value of the HL test for the simple model was 0.349 and 0.676 for the extended model.

An overview of the models’ performance (discriminative ability, AUC, and calibration, HL test) can be seen in Table 2.

**Table 2:** Overview of the results of the models’ performance

	Simple model	Extended model
Variables	11 main effect variables	13 main effect variables 10 bivariate interactions
AUC	0.789	0.790
HL test, p-value	0.3489	0.6764

## Discussion

### Principal findings

The objective of the study was to develop a predictive model that could identify persons with SMI and no contact with a GP using sociodemographic and clinical data collected from national registers. We developed two models: a simple prediction model with 11 variables and an extended prediction model with 13 variables and 10 two-way interactions. While a simple model may have better practical applicability [29], the extended model showed a slightly better performance. This was expected since the simple model was included in the expanded model. AUC showed slightly better predictive ability of the extended model, compared to the simple model. Though the p-value from the HL test was higher for the extended model compared to the simple model this does not necessarily mean that we can conclude a better calibration of the extended model. When testing the calibration of a model, that has been developed in a large dataset, in a smaller dataset, a lack of statistical significance can simply be due to the smaller sample size, and thus, lack of statistical power [33]. To deal with this issue, it has been suggested to assess the calibration curves’ deviation from the 45° line of perfect fit [33]. The calibration curves shown in Fig. 7 indicate greater conformity between the predicted probabilities and the observed probabilities for no contact with the GP in the extended model.

As depicted in the ROC curves, the threshold for when the predicted probability for no contact with GP is classified as the outcome is related to the model’s sensitivity and specificity [29]. In light of the aim of the study, we would like to argue for selecting a lower threshold. A low threshold permits the classification of persons at risk of not having contact with GP even when their predicted probabilities for no contact are relatively low. With a lower threshold we prioritize sensitivity over specificity as we consider the consequences of false negatives to be more severe than those of false positives, i.e., wrongly identifying a person as having regular contact with a GP, more severe than false positives, i.e., mistakenly identifying a person as having contact with a GP, whilst this is not true.

The sociodemographic variables predicting no contact with GP in our simple model, are generally in line with the existing literature. Similar to our study, young men were found to have a lower contact frequency with their GP in Germany and Australia [34, 35]. Although evidence is limited, ethnic minorities also seemed to have less access to healthcare services in accordance with the results [36]. Furthermore, people with SMI who are married or in a relationship have been documented to have more contact with GP than people with a single status [34, 37] Persons with SMI living in rural areas have limited access to healthcare often due to lack of transportation [36, 37]. This observation is likewise relevant to our study population, as the number of GPs per capita is lower in low population-density areas in Denmark [38]. The finding that the absence of comorbidity in persons with SMI yielded a higher risk of non-attendance with the GP, aligns with another Danish study showing higher contact with GPs among persons with schizophrenia and comorbidity as well as in an Australian study with people with psychotic disorders, where frequent attenders were more likely to suffer from chronic somatic health problems [11, 35]. The inclusion of interactions in the extended model improved the identification of persons with SMI and no contact with GP. To our knowledge, no studies in the literature have examined interactions between sociodemographic variables and contact with GP, making it difficult to discuss and compare our findings related to the extended prediction model.

In our study, we used *income*, *occupation*, and *education* as measures of socioeconomic position (SEP). In the simple model low income was associated with no contact with GP, as opposed to findings from Australia [35] and Latvia [39], where low SEP was associated with a higher contact frequency. In Denmark, it has been shown that people with a low SEP experience more barriers to accessing GP in general [40, 41]. Occupation and education only had a significant effect in the extended model.

In Denmark, it is possible to receive services from different healthcare sectors free of charge, which might influence the frequency of contacting a GP. Previously, secondary services have been considered when investigating contact with a GP among persons with schizophrenia in Denmark [11]. In Norway, continuity in contact with a GP was also assessed in relation to the use of out-of-hour services and acute hospital admissions among the general population [9]. In addition to sociodemographic factors, this study, therefore, analysed the effects of contact with different healthcare services on contact with GP. The results showed that contact with other healthcare services, such as secondary or psychiatric services, indeed can affect the contact frequency with a GP. This highlights the importance of considering multiple healthcare services, since not having contact with GP, does not necessarily indicate not having contact with all health care services.

### Strengths and limitations

A number of limitations in our study should be taken into account. We defined *no contact* as 0–2 contacts with GP in the follow-up period. This was because of the possibility of the GP reaching out to patients by telephone or email and registering this as a contact point in DPNR, even though no actual contact had been established. A cut-off for no contact at 0 contacts in the follow-up period, would entail a risk of misclassifying persons with SMI and no contact with GP as having contact. We considered it more important to capture all persons with SMI and possibly no contact with GP, than misclassifying some persons with few contacts (1–2 contacts in the follow-up period) as having no contact. There is a risk of misclassification of SMI due to its broad definition and complexity, although the DNPR-psych has almost complete coverage [18, 42], and the ICD-10 classification ensures standardization in diagnosis [43]. To differentiate between multiple diagnoses a hierarchy of SMI diagnoses based on their severity was created. However, misclassification within diagnoses might obscure or reduce the associations within each diagnostic group and contact with the GP.

Apart from misclassification, a risk of selection bias cannot be excluded. Recovery from diagnosis is not registered in the DNPR-Psych. As the inclusion period of the study consisted of five years, there is the potential of included persons with SMI recovering from their diagnosis during the inclusion period. Incorrect classification of SMI can impact the associations with contact with GP in the study population. Another risk of selection bias concerns persons with low functional capability due to SMI who might be too affected to seek health care and are therefore not registered with a diagnosis in the DNPR-Psych. If this is the case, the lack of inclusion of this group might reduce the associations with contact with GP further.

Comorbidity in the study population was estimated using a modified version of the Quality and Outcomes Framework (QOF). In the literature, the Charlson Comorbidity Index (CCI) has also been used [44]. QOF is a simpler measure than CCI, as it counts the number of comorbidities based on a list of frequent chronic diseases treated in GP and predicts variation in mortality in GP more effectively [28, 44]. However, we were only able to include diagnoses registered during hospital contacts, leaving out those comorbidities treated only in GP. The possibility of unmeasured comorbidity in the study population therefore exists. Moreover, there is a risk of confounding due to unmeasured variables. Loneliness and lifestyle factors in the literature were identified as influencing contact with GP among persons with mental illness [35, 45]. However, it was not possible to include these factors and the proxies for measuring these factors were also very limited. Therefore, these variables would not be possible to include in a register-based identification of persons with SMI and no contact with GP.

As our study population was identified using national registers the risk of selection bias in our study is minimal, thus making the study population representative for persons with SMI in Denmark. Representativity improves external validity, making the findings applicable to countries with comparable healthcare systems and similar populations [46]. The collection of data on contact to GPs in NHSR eliminated the risk of recall bias and GPs are financially incentivized to report all consultations, ultimately improving the completeness of the measurement of the variable [20]. Another strength of the study is the relatively long follow-up period. The 2-year follow-up period is comparable to other related studies [11, 34–37, 45, 47] and allows precise measurement of the outcome while maintaining the same exposure-outcome association over time [48].

### Implications

This study found the extended model optimal for the identification of persons with SMI and no contact with GP. While the models employed are relatively simple, logistic regression models, their performance is unlikely to be much improved from the use of more modern machine learning methods; the latter considerably increasing complexity.

The choice of the classification threshold for outcomes plays a pivotal role in determining the balance between false negatives and false positives in the identification. Consequently, it’s imperative to tailor the intervention strategy, considering factors such as invasiveness and associated costs. Further development and application will need to incorporate the perspectives of general practitioners and persons with SMI to validate and study the future use of such a model [49]. An existing intervention in Denmark focused on identifying somatic disease among persons with SMI [50], in which patient medical records were used for the identification of the target group. This identification method has been shown to be time-consuming, administratively heavy, and imprecise [50]. On a central level, the sociodemographic variables identified in this study can be used for developing a tool that physicians can use, potentially elevating the chances of reaching those persons with SMI, who do not have regular adequate contact with their GP.

## Conclusion

This study developed a prediction model to identify persons with SMI and no contact with GP using data obtained from Danish national registers. The model might be used in population programs focusing on prevention and timely treatment of somatic diseases, thereby possibly reducing premature mortality among persons with SMI. Developing a prediction tool based on sociodemographic variables can help physicians reach individuals who do not have regular contact with their GP. However, considerations of the optimal classification threshold for the model should be made, and future studies are needed to provide insight into the utility and effect of the prediction model in the identification of patients with SMI and no contact with a GP.

### Electronic supplementary material

Below is the link to the electronic supplementary material.

Supplementary Material 1

## Data Availability

The analysed data in the current study are not publicly available due to containing confidential individual information. Contact the corresponding author Maarten Rozing, mroz@sund.ku.dk for more information.

## References

[1] The Danish Health Technology Council. Behandlingsrådets rapport vedrørende ulighed i somatisk behandling af patienter med psykiske lidelser. The Danish Health Technology Council; 2023.

[2] World Health Organization. Helping people with severe mental disorders live longer and healthier lives Geneva. 2017. https://apps.who.int/iris/bitstream/handle/10665/259575/WHO-MSD-MER-17.7-eng. pdf?sequence = 1. (accessed March 1, 2023).

[3] World Health Organization. World mental health report l transforming mental health for all. World Health Organization; 2022.

[4] Crump C, Winkleby MA, Sundquist K, Sundquist J (2013). Comorbidities and mortality in persons with schizophrenia: a Swedish national cohort study. Am J Psychiatry.

[5] Heiberg IH, Jacobsen BK, Balteskard L, Bramness JG, Næss Ø, Ystrom E (2019). Undiagnosed cardiovascular disease prior to cardiovascular death in individuals with severe mental illness. Acta Psychiatr Scand.

[6] Rossom R, Hooker S, O’Connor P, Crain A, Sperl-Hillen J. Cardiovascular Risk for patients with and without Schizophrenia, Schizoaffective Disorder, or bipolar disorder. J Am Heart Assoc. 2022;11. 10.1161/JAHA.121.021444. 10.1161/JAHA.121.021444 PMC9075298 35261265

[7] Danish Health Data Authority. Beskrivelse af almen praksissektoren i Danmark. 2016.

[8] Danish Regions. Danske Regioner - Almen praksis. Dan Reg 2023. https://www.regioner.dk/sundhed/praksissektoren/almen-praksis (accessed March 3, 2023).

[9] Sandvik H, Hetlevik Ø, Blinkenberg J, Hunskaar S (2022). Continuity in general practice as predictor of mortality, acute hospitalisation, and use of out-of-hours care: a registry-based observational study in Norway. Br J Gen Pract.

[10] Larkin J, Pericin I, Osborne B, Dodd P, Collins C (2022). Cross-sectional analysis of coding, patient characteristics, consultation frequency and pharmacological treatment of adults with severe mental disorders in Irish general practice. Ir J Med Sci.

[11] Nørgaard H, Schou Pedersen H, Fenger-Grøn M, Vestergaard M, Nordentoft M, Laursen T (2019). Schizophrenia and attendance in primary healthcare: a population-based matched cohort study. Scand J Prim Health Care.

[12] Tusa N, Koponen H, Kautiainen H, Korniloff K, Raatikainen I, Elfving P (2019). The profiles of health care utilization among a non-depressed population and patients with depressive symptoms with and without clinical depression. Scand J Prim Health Care.

[13] Bower P, Grigoroglou C, Anselmi L, Kontopantelis E, Sutton M, Ashworth M (2020). Is health research undertaken where the burden of disease is greatest? Observational study of geographical inequalities in recruitment to research in England 2013–2018. BMC Med.

[14] Tranberg K, Due TD, Jønsson ABR, Kousgaard M, Rozing M, Møller A. (In press). Challenges in reaching patients with severe illness for trials in general practice—a convergent mixed methods study based on the SOFIA pilot trial. BMC Pilot Feasibility Stud 2023. 10.1186/s40814-023-01395-y PMC10617218 37908003

[15] Rozing MP, Jønsson A, Køster-Rasmussen R, Due TD, Brodersen J, Bissenbakker KH (2021). The SOFIA pilot trial: a cluster-randomized trial of coordinated, co-produced care to reduce mortality and improve quality of life in people with severe mental illness in the general practice setting. Pilot Feasibility Stud.

[16] Van Calster B, Wynants L, Timmerman D, Steyerberg EW, Collins GS (2019). Predictive analytics in health care: how can we know it works?. J Am Med Inf Assoc.

[17] Zhao Y, Wood EP, Mirin N, Cook SH, Chunara R (2021). Social determinants in Machine Learning Cardiovascular Disease Prediction models: a systematic review. Am J Prev Med.

[18] Mors O, Perto GP, Mortensen PB (2011). The Danish Psychiatric Central Research Register. Scand J Public Health.

[19] Schmidt M, Schmidt SAJ, Sandegaard JL, Ehrenstein V, Pedersen L, Sørensen HT (2015). The Danish National Patient Registry: a review of content, data quality, and research potential. Clin Epidemiol.

[20] Sahl Andersen J, De Fine Olivarius N, Krasnik A (2011). The Danish National Health Service Register. Scand J Public Health.

[21] The Danish National Archives. Det Centrale Personregister / Den Ny Kirkebog 2023. 10.5279/DK-RA-19392.

[22] Danmarks Statistik. TIMES opgave - Forebyggelsesregistret 2023. https://www.dst.dk/da/Statistik/dokumentation/Times/forebyggelsesregistret (accessed April 10, 2023).

[23] Danmarks Statistik. Registerdeklaration for Dødsårsagsregisteret. 2013.

[24] Danmarks Statistik. Personers tilknytning til arbejdsmarkedet set over hele året (AKM) 2023. https://www.dst.dk/da/TilSalg/Forskningsservice/Dokumentation/hoejkvalitetsvariable/personers-tilknytning-til-arbejdsmarkedet-set-over-hele-aaret--akm (accessed March 29, 2023).

[25] Baadsgaard M, Quitzau J (2011). Danish registers on personal income and transfer payments. Scand J Public Health.

[26] Jensen VM, Rasmussen AW (2011). Danish education registers. Scand J Public Health.

[27] World Health Organization. ICD-10 Version:2019. IcdWhoInt 2019. https://icd.who.int/browse10/2019/en (accessed August 24, 2023).

[28] Brilleman SL, Gravelle H, Hollinghurst S, Purdy S, Salisbury C, Windmeijer F (2014). Keep it simple? Predicting primary health care costs with clinical morbidity measures. J Health Econ.

[29] Verbakel JY, Steyerberg EW, Uno H, De Cock B, Wynants L, Collins GS (2020). ROC curves for clinical prediction models part 1. ROC plots showed no added value above the AUC when evaluating the performance of clinical prediction models. J Clin Epidemiol.

[30] Narkhede S, Understanding. AUC - ROC Curve. Data Sci 2018. https://towardsdatascience.com/understanding-auc-roc-curve-68b2303cc9c5 (accessed April 24, 2023).

[31] Fagerland MW, Hosmer DW (2012). A generalized Hosmer–lemeshow goodness-of-fit test for Multinomial Logistic Regression models. Stata J Promot Commun Stat Stata.

[32] Kreiner S (2018). Statistisk problemløsning: præmisser, teknik og analyse. 2. Udgave. 2. Oplag.

[33] Kramer AA, Zimmerman JE (2007). Assessing the calibration of mortality benchmarks in critical care: the Hosmer-Lemeshow test revisited*. Crit Care Med.

[34] Tomczyk S, Schomerus G, Stolzenburg S, Muehlan H, Schmidt S (2018). Who is seeking whom? A person-centred approach to help-seeking in adults with currently untreated mental health problems via latent class analysis. Soc Psychiatry Psychiatr Epidemiol.

[35] Waterreus A, Morgan V (2018). Treating body, treating mind: the experiences of people with psychotic disorders and their general practitioners - findings from the Australian National Survey of High Impact psychosis. Aust N Z J Psychiatry.

[36] Kroll D, Latham C, Mahal J, Siciliano M, Shea L, Irwin L (2019). A successful Walk-In Psychiatric Model for Integrated Care. J Am Board Fam Med.

[37] Castillejos M, Martín-Pérez C, Mayoral-Cleries F, Bordallo-Aragón A, Sepúlveda-Muñoz J, Moreno-Küstner B (2018). Factors associated with visits to general practitioners in patients with schizophrenia in Malaga. BMC Fam Pr.

[38] The Danish Organization of General Practitioners. En styrket praksissektor– vejen til mindre ulighed i sundhed. Sjællandske Medier 2023.

[39] Rancans E, Renemane L, Kivite-Urtane A, Ziedonis D. Prevalence and associated factors of mental disorders in the nationwide primary care population in Latvia: a cross-sectional study. Ann Gen Psychiatry. 2020;19. 10.1186/s12991-020-00276-5. 10.1186/s12991-020-00276-5 PMC7137231 32280360

[40] National Institute of Public Health. Social ulighed i mødet med sundhedsvæsenet: en systematisk litteraturgennemgang. Danish Health Authority; 2022.

[41] Pedersen PV. Socialt udsattes møde med sundhedsvæsenet. Statens Institut for Folkesundhed, SDU; 2019.

[42] Jakobsen KD, Frederiksen JN, Hansen T, Jansson LB, Parnas J, Werge T (2005). Reliability of clinical ICD-10 schizophrenia diagnoses. Nord J Psychiatry.

[43] Nickelsen TN. Datavaliditet og dækningsgrad i Landspatientregisteret. Ugeskriftet.dk 2005. 11810794

[44] Carey IM, Shah SM, Harris T, DeWilde S, Cook DG (2013). A new simple primary care morbidity score predicted mortality and better explains between practice variations than the Charlson index. J Clin Epidemiol.

[45] Gervaix J, Haour G, Michel M, Chevreul K (2019). Impact of mental illness on care for somatic comorbidities in France: a nation-wide hospital-based observational study. Epidemiol Psychiatr Sci.

[46] Song JW, Chung KC (2010). Observational studies: Cohort and Case-Control studies. Plast Reconstr Surg.

[47] Lankila T, Laatikainen T, Wikström K, Linna M, Antikainen H (2022). Association of travel time with mental health service use in primary health care according to contact type - a register-based study in Kainuu, Finland. BMC Health Serv Res.

[48] Sedgwick P (2014). Retrospective cohort studies: advantages and disadvantages. BMJ.

[49] McNemar E. What Are the Benefits of Predictive Analytics in Healthcare? HealthITAnalytics. 2021. https://healthitanalytics.com/news/what-are-the-benefits-of-predictive-analytics-in-healthcare (accessed May 24, 2023).

[50] Tranberg K, Søndergaard E, Nielsen MH, Møller A. Ydelse 2150– et redskab til at mindske ulighed i sundhed i almen praksis? Månedsskr Almen PRaksis 2023.

[51] Retsinformation D. Retsinformation. 2018. http://www.retsinformation.dk/eli/lta/2018/502 (accessed October 23, 2023).

