# Supplementary material for: Developing a prediction model to identify people with severe mental illness without regular contact to their GP - a study based on data from the Danish national registers
Source: BMC Psychiatry. 2024 Apr 23;24:301. doi: 10.1186/s12888-024-05743-x (PMC11040846; doi:10.1186/s12888-024-05743-x)
Supplement: Supplementary file 1 — Supplementary Material 1 [file 12888_2024_5743_MOESM1_ESM.docx]

# Supplementary materials

Supplementary file 1

File format: .pdf

Title of data: Modified version of the Quality and Outcomes Framework (QOF)*

Description: An overview of the conditions and ICD-codings from the Quality and Outcomes Framework included/not included in the variable for *Comorbidity.*

Supplementary file 2

File format: .pdf

Title of data: List of included variables

Description: List of included variables characterizing the study population and used in the descriptive and statistical analyses.

Supplementary file 3

File format: .pdf

Title of data: Contact to GP in the follow-up period

Description: A multi panel figure showing Contact to general practice in each diagnostic group and Contact to general practice in the study population between 2016-2017.

Supplementary file 4

File format: .pdf

Title of data: The simple prediction model

Description: A table of the simple prediction model. The model is a logistic regression containing 11 variables on contact to general practice among persons with severe mental illness. The table contains information on odd-ratios, confidence intervals and p-values.

## Modified version of the Quality and Outcomes Framework (QOF)*

| QOF Condition | ICD-coding | Included/not included in *Comorbidity* |
| --- | --- | --- |
| Asthma | **J45** Asthma  **J46** Status asthmaticus | Included |
| Atrial fibrillation | **I48** Atrial fibrillation and flutter | Included |
| Cancer (excluding non-melanotic skin cancers) | **C00-C96** Malignant neoplasms (excluding **C44** Other malignant neoplasms of skin) | Included |
| Chronic kidney disease (stages 3-5) | **N18** Chronic Kidney Disease (excluding **N18.1-2** Chronic kidney disease, stages 1-2) | Included |
| COPD | **J41** Simple and mucopurulent chronic bronchitis  **J42** Unspecified chronic bronchitis  **J43** Emphysema  **J44** Chronic obstructive pulmonary disease | Included |
| Coronary heart disease | **I20-I25** Ischemic heart disease (excluding **I20.1** Angina pectoris with documented spasm,  **I25.3** Aneurysm of heart,  **I25.4** Coronary artery aneurysm and dissection) | Included |
| Dementia | **G30** Alzheimer's disease  **G31** Other degenerative diseases of nervous system, not elsewhere classified **F00** Dementia in Alzheimer's disease  **F01** Vascular dementia  **F02** Dementia in other diseases classified elsewhere  **F03** Unspecified dementia | Included |
| Depression | **F20.4** Post-schizophrenic depression  **F25.1** Schizoaffective disorder, depressive type  **F32** Major depressive disorder, single episode  **F33** Major depressive disorder, recurrent **F34.1** Dysthymia  **F41.2** Mixed anxiety and depressive disorder | Not included |
| Diabetes | **E10** Type 1 diabetes mellitus  **E11** Type 2 diabetes mellitus  **E12** Malnutrition-related diabetes mellitus **E13** Other specified diabetes mellitus  **E14** Unspecified diabetes mellitus | Included |
| Epilepsy | **G40** Epilepsy and recurrent seizures (excluding **G40.4** Other generalized epilepsy and epileptic syndromes)  **G41** Status epilepticus | Included |
| Heart Failure | **I11.0** Hypertensive heart disease with (congestive) heart failure  **I13.0** Hypertensive heart and renal disease with (congestive) heart failure  **I13.2** Hypertensive heart and renal disease with both (congestive) heart failure and renal failure  **I50** Heart failure | Included |
| Hypertension | **I10** Essential (primary) hypertension  **I11** Hypertensive heart disease  **I12** Hypertensive renal disease  **I13** Hypertensive heart and renal disease **I15** Secondary hypertension | Included |
| Hypothyroidism | **E00** Congenital iodine-deficiency syndrome **E01** Iodine-deficiency related thyroid disorders and allied conditions  **E02** Subclinical iodine-deficiency hypothyroidism  **E03** Other hypothyroidism  **E89.0** Postprocedural hypothyroidism | Included |
| Psychosis, schizophrenia + bipolar affective disorders | **F20** Schizophrenia  **F22** Delusional disorders  **F23** Brief psychotic disorder  **F24** Induced delusional disorder  **F25** Schizoaffective disorders  **F28** Other psychotic disorder not due to a substance or known physiological condition **F29** Unspecified psychosis not due to a substance or known physiological condition **F30** Manic episode  **F31** Bipolar disorder  **F32.3** Severe depressive episode with psychotic symptoms  **F33.3** Recurrent depressive disorder, current episode severe with psychotic symptoms | Not included |
| Stroke or TIA | **G45** Transient cerebral ischemic attacks and related syndromes  **I61** Nontraumatic intracerebral hemorrhage  **I63** Cerebral infarction  **I64** Stroke, not specified as haemorrhage or infarction | Included |

*The list has been taken from Carey et al. 2013. *Dementia without other symptoms* and *Alzheimer's* have been added to the list [[43]](https://www.zotero.org/google-docs/?fr5dCI).

## List of included variables

| **Variable** | **Categories** |
| --- | --- |
| **Severe mental illness (SMI)** | Severe depression |
|  | Bipolar illness |
|  | Psychotic illness |
| **Age** | 18-29 years |
|  | 30-39 years |
|  | 40-49 years |
|  | 50-59 years |
|  | 60-69 years |
|  | 70+ years |
| **Sex** | Male |
|  | Female |
| **Region** | Northern Jutland |
|  | Mid Jutland |
|  | Southern Denmark |
|  | Capitol City |
|  | Zealand |
| **Ethnicity** | Danish |
|  | Immigrants and  descendants |
| **Marital status** | Married/in a relationship |
|  | Not married |
|  | Divorced |
|  | Widow |
| **Occupation** | Working |
|  | Without work |
|  | Retired |
| **Income** | <150,000 DKK. |
|  | 150,001-225,000 DKK. |
|  | 225,001-300,000 DKK. |
|  | >300,000 DKK. |
| **Education** | Short |
|  | Intermediate |
|  | Long |
| **Comorbidity** | 0 |
|  | 1 |
|  | ≧2 |
| **Contact with general practice** | Contact (>2) |
|  | No contact (≤2) |
| **Out-of-hours contact** | 0 |
|  | 1-2 |
|  | 3-6 |
|  | ≧7 |
| **Contacts to secondary services (hospital)** | 0 |
|  | 1-2 |
|  | 3-6 |
|  | ≧7 |
| **Contact to psychiatric services** | 0 |
|  | 1-2 |
|  | 3-6 |
|  | ≧7 |

## Contact to GP in the follow-up period


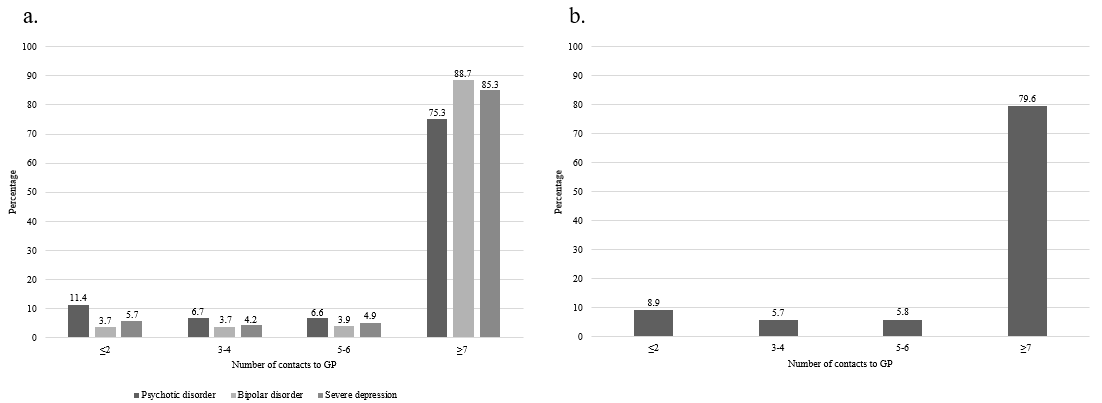


- 1. Contact to GP in each diagnostic group
  2. Contact to GP in the study population

1. ***The simple prediction model***

|  | OR for no contact (≤2) with GP [95% CI] | p-value |
| --- | --- | --- |
| Severe mental illness | | **<0.0001*** |
| Severe depression | 1 | - |
| Psychotic disease | 1.39 [1.22;1.58] | <0.0001* |
| Bipolar disease | 0.69 [0,58;0.80] | <0.0001* |
| Age | | **<0.0001*** |
| 18-29 years | 1 | - |
| 30-39 years | 1.07 [0.97;1.19] | 0.1687 |
| 40-49 years | 1.03 [0.93;1.15] | 0.5459 |
| 50-59 years | 0.93 [0.82;1.05] | 0.2591 |
| 60-69 years | 0.94 [0.80;1.10] | 0.4227 |
| 70+ years | 0.58 [0,45;0.75] | <0.0001* |
| Sex | | **<0.0001*** |
| Man | 1 | - |
| Female | 0.36 [0.33;0.39] | <0.0001* |
| Region | | **<0.0001*** |
| North Jutland Region | 1 | - |
| Mid Jutland Region | 0.46 [0.38;0.54] | <0.0001* |
| Region of Southern Denmark | 1.06 [0.90;1.25] | 0.4721 |
| Capital City Region | 1.38 [1,18;1,61] | <0.0001* |
| Region Zealand | 1.55 [1,31;1.84] | <0.0001* |
| Ethnicity | | **<0.0001*** |
| Danish | 1 | - |
| Non-Danish | 1.32 [1.20;1.45] | <0.0001* |
| Marital status | | **<0.0001*** |
| Married/in a relationship | 1 | - |
| Unmarried | 1.54 [1.36;1.75] | <0.0001* |
| Divorced | 1.30 [1.12;1.51] | 0.0005* |
| Widow | 1.35 [0.96;1.90] | 0.0809 |
| Income | | **<0.0001*** |
| <150000 DKK. | 1 | - |
| 150001-225000 DKK. | 0.82 [0.75;0.89] | <0.0001* |
| 225001-300000 DKK. | 0.66 [0.57;0.76] | <0.0001* |
| >300000 DKK. | 1.00 [0.84;1.18] | 0.9619 |
| Comorbidity | | **<0.0001*** |
| 0 | 1 | - |
| 1 | 0.57 [0.50;0.65] | <0.0001* |
| ≧2 | 0.28 [0.21;0.39] | <0.0001* |
| Out-of-hours contacts | | **<0.0001*** |
| 0 | 1 | - |
| 1-2 | 0.50 [0.43;0.57] | <0.0001* |
| 3-6 | 0.37 [0.31;0.45] | <0.0001* |
| ≧7 | 0.17 [0.13;0.23] | <0.0001* |
| Contact with secondary services (hospital) | | **<0.0001*** |
| 0 | 1 | - |
| 1-2 | 0.41 [0.37;0.45] | <0.0001* |
| 3-6 | 0.27 [0.24;0.30] | <0.0001* |
| ≧7 | 0.22 [0.19;0.26] | <0.0001* |
| Contact with psychiatric services | | **<0.0001*** |
| 0 | 1 | - |
| 1-2 | 0.82 [0.75;0.89] | <0.0001* |
| 3-6 | 0.95 [0.85;1.07] | 0.3947 |
| ≧7 | 1.19 [1.02;1.38] | 0.0309 |
| *1% significance level |  |  |
